# Targeting Autophagy in Ovarian Cancer: The Emerging Role of Ginsenosides

**DOI:** 10.1002/cam4.71717

**Published:** 2026-03-11

**Authors:** Yuxin Guo, Cuilan Yun, Yuemei Zhang, Xu Yang, Huiqin Liu

**Affiliations:** ^1^ Department of Obstetrics and Gynecology Affiliated Baotou Central Hospital of Baotou Medical College Baotou China; ^2^ Department of Obstetrics and Gynecology Baotou Central Hospital Baotou China

**Keywords:** autophagy, ginsenosides, mTOR, ovarian cancer

## Abstract

**Background:**

Ovarian cancer, the third most prevalent gynecological malignancy, is frequently diagnosed at an advanced stage owing to its asymptomatic early progression. Despite the application of conventional therapies, clinical management remains limited by adverse effects and the development of drug resistance. Therefore, the identification of novel therapeutic targets and strategies is urgently needed. Autophagy, a tightly regulated cellular degradation process, plays a dual and context‐dependent role in cancer progression and chemoresistance and has emerged as a promising therapeutic target in ovarian cancer. Ginsenosides, the major bioactive constituents of ginseng, exhibit significant anticancer activity in a variety of tumors.

**Methods:**

A literature review was conducted to summarize current studies on autophagy regulation in ovarian cancer and the structural characteristics and pharmacological activities of ginsenosides, with particular attention to the molecular mechanisms through which ginsenosides modulate autophagy and their potential therapeutic implications in ovarian cancer.

**Results:**

Ginsenosides can modulate autophagy through multiple mechanisms, including activation of the AMPK/mTOR signaling pathway, induction of reactive oxygen species (ROS) accumulation, and regulation of autophagy‐related genes (ATGs), ultimately contributing to tumor suppression. Moreover, ginsenosides have demonstrated notable anticancer effects in ovarian cancer, further highlighting their potential clinical value.

**Conclusion:**

This review provides a comprehensive overview of current knowledge regarding autophagy regulation in ovarian cancer, summarizes the structural and pharmacological characteristics of ginsenosides, and discusses their emerging role as autophagy‐targeting agents, particularly in the treatment of this malignancy. Collectively, these insights offer a new perspective for the development of autophagy‐based precision therapies for ovarian cancer.

## 1 Introduction

Ovarian cancer is the most lethal malignancy among tumors of the female reproductive system, and approximately 70% of patients are diagnosed at an advanced stage, with a 5‐year survival rate of less than 40% [1, 2]. However, currently available treatments have shown limited efficacy in improving the prognosis of ovarian cancer patients. Recent studies indicate that even with the addition of novel gene‐targeted therapies to first‐line chemotherapeutic regimens, the overall survival remains below 15 months [3, 4]. Therefore, it is imperative to investigate new therapeutic approaches. Emerging evidence suggests that modulation of autophagy can be a promising approach to inhibiting cancer progression, overcoming chemoresistance, and optimizing therapeutic responses in ovarian cancer [5, 6].

Autophagy is a highly conserved cellular metabolic process that plays a dual role in cancer progression, either promoting or inhibiting tumor development depending on the stage of the disease. It influences the growth, metastasis, and invasion of cancer cells through modulation of the tumor microenvironment, regulation of intracellular homeostasis, and interaction with oncogenic signaling pathways [7]. By efficiently eliminating chemotherapy‐induced cellular damage, suppressing immune responses, and supplying metabolic substrates, autophagy has also become a key mechanism contributing to cancer drug resistance [8]. Accordingly, autophagy has emerged as a critical pharmacological target for the treatment of ovarian cancer.

In recent years, the role of natural compounds in inhibiting ovarian cancer by modulating autophagy has gradually gained attention [9]. Ginseng is a highly valued traditional Chinese medicinal herb, and its primary active components, ginsenosides, have demonstrated potent anticancer activities across various types of cancer [10]. Compared with traditional chemotherapeutic drugs, ginsenosides exhibit favorable pharmacological activities and can exert anti‐tumor effects through various mechanisms, such as inducing autophagy and apoptosis, regulating the cell cycle, etc., significantly inhibiting the progression of cancer [11, 12]. Therefore, ginsenosides may represent a promising natural antitumor agent to enhance current therapies and improve outcomes in ovarian cancer. This review comprehensively outlines the role of autophagy in ovarian cancer progression, delineates the mechanisms by which ginsenosides regulate autophagy in cancer, and discusses their therapeutic potential in ovarian cancer treatment, thereby offering new perspectives for clinical management.

## 2 Ovarian Cancer: Staging, Risk Factors, and Treatment

### 2.1 Epidemiology and Classification of Ovarian Cancer

Ovarian cancer is most common in women aged 55–59 years, and it is projected that by 2040, there will be more than 400,000 new cases worldwide, with a mortality rate of approximately 75% [2, 13]. Early diagnosis and pathologic staging are critical and will directly affect postoperative survival [14]. Ovarian tumors are generally classified into three major types: epithelial, germ cell, and sex cord‐stromal tumors [15]. Epithelial ovarian cancer is the most common and lethal type, accounting for more than 90% of cases, and is mainly categorized into high‐grade serous ovarian carcinoma (HGSOC), low‐grade serous ovarian carcinoma (LGSOC), mucinous ovarian carcinoma (MOC), endometrioid ovarian carcinoma (EOVC), and ovarian clear cell carcinoma (OCCC) [15, 16]. The most common type, HGSOC, with high chromosomal instability, extensive TP53 mutations, and poor prognosis, may originate from Fallopian tube epithelium (FTE) or preciliated cells [17, 18]. LGSOC has a low rate of TP53 mutations, slow progression, and a better early prognosis [19]. MOC is associated with a favorable early prognosis and is particularly common in young women [20]. EOVC is associated with endometrial disease and is often comorbid with endometrial cancer [21]. OCCC has a worse prognosis and is less sensitive to chemotherapy [22]. Ovarian germ cell tumors (OGCTs) originate from primordial germ cells and primarily affect women under the age of 30. They are highly sensitive to platinum‐based chemotherapy and generally have a favorable prognosis [23]. Ovarian sex cord‐stromal tumors (SCSTs) are relatively rare, accounting for approximately 5%–8% of all malignant ovarian tumors. The most common subtype is the granulosa cell tumor, which generally has a better prognosis than epithelial ovarian cancer [24]. An improved understanding of these subtypes facilitates personalized and targeted therapy.

### 2.2 Risk Factors and Prevention of Ovarian Cancer

Ovarian cancer development is associated with a variety of risk factors, such as genetic predisposition, hormonal status, reproductive history, and lifestyle‐related influences. High‐risk factors for the development of ovarian cancer include mutations in the Breast Cancer gene 1 (BRCA1) or Breast Cancer gene 2 (BRCA2) genes, early menarche or late menopause, infertility, and prolonged hormone therapy [25, 26, 27, 28, 29, 30, 31]. Insulin secretion rate, smoking, obesity, diseases related to the female reproductive system, and pelvic inflammatory diseases are also associated with ovarian cancer [32, 33, 34, 35, 36, 37]. In contrast, ovarian cancer risk can be reduced by oral contraceptives, pregnancy, breast‐feeding, and female reproductive system surgery [38, 39, 40, 41, 42]. Dietary habits also play an important role. Vegetables, dietary fiber, and green tea are associated with a reduced risk, while saturated fats, animal fats, and nitrites have been linked to an increased risk [43]. Understanding these factors enables early screening and prevention of ovarian cancer and may provide guidance for subsequent treatment (Table 1).

**TABLE 1:** Factors associated with the development of ovarian cancer.

Form	Type of factor	Specific factors	Impact description	References
Genetic factor	Risk factor	BRCA1/BRCA2 gene mutations	BRCA1 or BRCA2 carriers have 1–4 times the risk of developing ovarian cancer than non‐BRCA carriers, and BRCA1 carriers have about 3 times the risk of BRCA2 carriers.	[26]
Hormone‐related	Risk factor	Early onset of menstruation or late menopause	Increased risk of disease due to prolonged estrogen action, prolonged lifetime ovulation years (LOY), and increased number of ovulations.	[27, 28]
Hormone‐related	Risk factor	Female infertility	Significantly associated with the development of ovarian cancer.	[29]
Hormone‐related	Risk factor	Assisted Reproductive Technology (ART)	Increased risk of ovarian cancer, mainly due to infertility itself.	[30]
Hormone‐related	Risk factor	Menopausal Hormone Therapy (MHT)	Long‐term use significantly increases ovarian cancer risk.	[31]
Hormone‐related	Risk factor	Hormone replacement therapy (HRT)	Use of monoestrogen therapy for more than 10 years is associated with a significant increase in the risk of disease, and continuous estrogen‐progesterone replacement therapy (EPRT) is recommended as a preferred option.	[25]
Metabolism‐related	Risk factor	Decreased insulin secretion rate	Causal effect with the high risk of developing ovarian cancer.	[33]
Lifestyle	Risk factor	Cigarette smoking	Early exposure to smoking increases the risk of ovarian cancer in adulthood.	[34]
Lifestyle	Risk factor	Obesity	Promoting tumor metastasis and adhesion by inducing an inflammatory response is positively associated with ovarian cancer risk.	[35, 36]
Gynecological diseases	Risk factor	Diseases related to the female reproductive system	For example, endometriosis and adenomyosis were significantly associated with the risk of EOVC and OCCC.	[37]
Gynecological diseases	Risk factor	Pelvic inflammatory disease	May increase the risk of epithelial ovarian cancer by mediating inflammatory response	[32]
Fertility‐related	Protective factor	Oral contraceptive	Especially for BRCA1 carriers, the protective effect is time‐dependent and lasts for many years after discontinuation of the drug.	[39, 40]
Fertility‐related	Protective factor	Pregnancies	The protective effect was mainly associated with the first three pregnancies, which reduced the risk of ovarian cancer by 21%, 26%, and 12%, respectively, and included incomplete pregnancies (e.g., miscarriages).	[41, 44]
Fertility‐related	Protective factor	Nursing	It is an independent protective factor against ovarian cancer, and longer nursing is associated with lower risk.	[42]
Surgical interventions	Protective factor	Surgery related to the female reproductive system	Hysterectomy, tubal ligation or resection significantly reduces the risk of ovarian cancer regardless of a family history of ovarian cancer.	[38]
Dietary	Protective factor	High vegetable/fiber/green tea intake	Significantly reduces the risk of ovarian cancer	[43]
Dietary	Risk factor	Saturated fat, animal fat, nitrite intake	Increased risk of ovarian cancer	[43]

### 2.3 Current Treatment Strategies for Ovarian Cancer

Currently, surgery and chemotherapy are the main treatments for ovarian cancer. Surgery includes comprehensive staging surgery and tumor debulking surgery. For patients with early‐stage epithelial ovarian cancer, comprehensive staging surgery is highly beneficial, but there is a significant risk of intraoperative or postoperative problems, and more than 30% of patients will be left with long‐term sequelae [45, 46]. For patients with advanced disease who have the possibility of achieving complete tumor reduction, primary tumor cytoreduction is performed; otherwise, neoadjuvant chemotherapy is administered first. The optimal course of treatment for patients who have no chance of surgery is chemotherapy. The standard first‐line treatment regimen is combination chemotherapy based on platinum‐based drugs combined with paclitaxel. However, the majority of patients will develop resistance to chemotherapeutic agents, and the efficacy of treatment is substantially reduced [47, 48]. The mechanism of drug resistance may be related to altered DNA damage repair (DDR) pathways, dysregulation of cell cycle regulation, and inhibition of intracellular metabolism and apoptosis [49]. As research into these mechanisms advances, novel therapeutic approaches such as immunotherapy and molecular‐targeted therapy have emerged [50]. Unfortunately, numerous clinical trials have shown that most of these new strategies fail to significantly improve clinical outcomes, with the progression‐free survival in drug‐resistant cases remaining less than four months [51]. Their efficacy still requires further validation.

### 2.4 Challenges and Future Prospects in Ovarian Cancer Treatment

In summary, ovarian cancer treatment is confronted with two major obstacles. First, while surgery remains effective, its associated complications often reduce patients' quality of life, and it is not feasible for those diagnosed at advanced stages. Second, chemoresistance significantly worsens clinical outcomes, and the therapeutic benefits of novel treatments remain to be fully established. Consequently, the overall survival rate of ovarian cancer has shown only modest improvement [52]. Therefore, there is an urgent need to explore innovative therapeutic strategies. In this context, the complex role of autophagy in cancer development and therapy has attracted growing attention.

## 3 Autophagy in Ovarian Cancer

Autophagy is a crucial mechanism by which cells respond to stress, mediating processes such as cell survival, death, and organelle clearance in response to external stress [53]. A basal level of autophagy normally exists within cells to support growth, development, and homeostasis; however, when cells are exposed to stress, autophagic flux increases markedly [54, 55].

### 3.1 Definition, Classification, and Mechanisms

As an intracellular degradation and recycling process, autophagy sustains intracellular homeostasis by transporting harmful substances or damaged organelles from the cytoplasm to the lysosome for degradation, removal, and recycling [56, 57]. In mammalian cells, it can be categorized as macroautophagy, microautophagy, and chaperone‐mediated autophagy (CMA) according to the mode of substrate entry into the lysosome [58]. Macroautophagy is the most dominant and well‐known form of autophagy. The initiation depends on the production of the UNC‐51‐like kinase 1 complex (ULK1 complex), including UNC‐51‐like kinase 1 (ULK1), AK family kinase‐interacting protein of 200 kDa (FIP200), Autophagy‐related protein 13 (ATG13), and Autophagy‐related protein 101 (ATG101), which will activate the Phosphoinositide 3‐Kinase complex (PI3K complex), including Bcl‐2 interacting protein 1 (Beclin1), Vacuolar Protein Sorting 34 (VPS34), Vacuolar Protein Sorting 15 (VPS15), and Autophagy‐related protein 14‐like (ATG14L), thereby inducing the process of nucleation and the formation of autophagy precursors [59]. Autophagy‐related protein 4 (ATG4) processes the Autophagy‐related protein 8 (ATG8) family—known as microtubule‐associated protein 1 light chain 3 (LC3) in mammalian cells—into LC3‐I. Subsequently, Autophagy‐related protein 7 (ATG7) and Autophagy‐related protein 3 (ATG3) mediate the lipidation reaction to generate LC3‐II. The Autophagy‐related protein 5‐Autophagy‐related protein 12‐Autophagy‐related protein 16 complex (Atg5‐Atg12‐Atg16 complex) acts as an E3 ubiquitin‐protein ligase‐like enzyme (E3 enzyme), anchoring it to the autophagosome membrane, facilitating fusion and expansion of the membrane, and encircling the degradation components to form an intact autophagosome [60]. Subsequently, the autophagosome moves along microtubules toward the perinuclear region, where the SNARE (Soluble NSF Attachment Protein REceptor) protein complex and Ras‐related protein Rab‐7 (Rab7) will drive the fusion of the two membranes with each other to form an autophagolysosome [61, 62]. Eventually, biomolecules are broken down into small molecules by hydrolytic enzymes under acidic conditions within the autolysosome. These molecules are then released into the cytoplasm for cellular reuse via transporter proteins [62, 63]. Lysosomes wrap substrates directly through membrane protrusion and invagination in microautophagy [58]. CMA can only recognize substrates with KFERQ (Lys‐Phe‐Glu‐Arg‐Gln) sequences [64].

**FIGURE 1:**
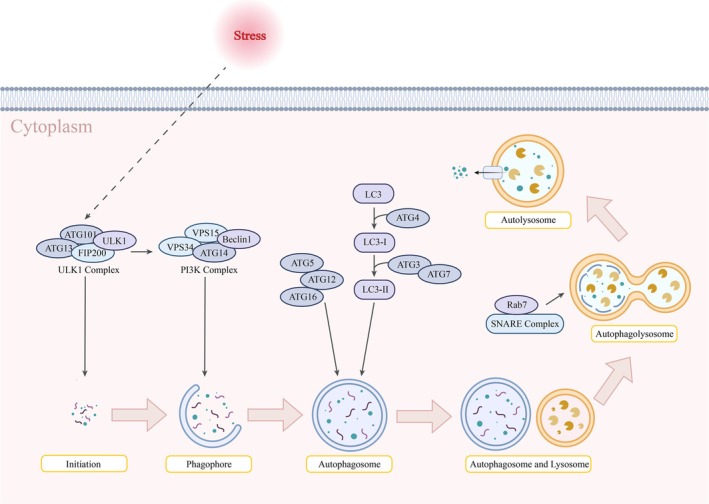
Autophagy process schematic diagram. Autophagy can be triggered by stress. The ULK1 complex is necessary for its initiation, activating the PI3K complex to induce nucleation and formation of autophagy precursors. Atg4, Atg3, and Atg7 contribute to the generation of LC3‐II, and the Atg12‐Atg5‐Atg16 complex assists in its localization and promotes autophagosome formation. It then moves to the area around the lysosome, where the SNARE protein complex and Rab7 drive the fusion of the two membranes to form the autophagolysosome. Eventually, biomolecules are degraded within the autolysosome and released into the cytoplasm for cellular reuse.

Depending on whether it can identify and degrade the substrate in a specific way, it can also be classified into two groups: selective autophagy and non‐selective autophagy. Selective autophagy is prevalent in cells and is capable of removing dysfunctional or excessive mitochondria, endoplasmic reticulum, ribosomes, etc., [65, 66, 67]. Non‐selective autophagy can degrade non‐specific substances to provide essential energy under stress [68]. The previously mentioned autophagic processes interact and regulate each other in the cell and play different roles at different stages of disease development (Figure 1) [69].

### 3.2 The Role of Autophagy in Cancer

Autophagy has a complex role in cancer. On the one hand, autophagy is an essential mechanism for maintaining normal cellular function; on the other hand, cancer progression to advanced stages usually depends on autophagic activity [70]. It can be both protective and pathogenic in cancer, but one role usually predominates at a given stage or under specific conditions [71].

On the tumor‐suppressive side, it serves as a principal cellular process responsible for sustaining cellular integrity, redox homeostasis, and protein stability [7]. By eliminating damaged organelles and proteins, autophagy maintains genomic stability in the early stages of cancer, inhibiting its progression [72]. Mechanistically, autophagy suppresses tumor initiation through activation of the ULK1 complex and downstream formation of autophagosomes via the Beclin1–Vps34 complex, which facilitates the removal of reactive oxygen species (ROS) and misfolded proteins [73]. This protective function helps maintain genomic stability and prevents malignant transformation. Conversely, when autophagy is impaired, the accumulation of ROS and damaged organelles promotes DNA damage and oncogenic mutations, thereby increasing the risk of tumorigenesis [74, 75]. Impaired autophagy increases the ability of cancer cells to invade and proliferate, facilitates tumor growth even at advanced stages, and influences tumor invasion and metastasis by controlling the transformation from epithelial to mesenchymal(EMT) [76, 77, 78]. Therefore, loss of autophagic function increases the potential of cancer cells to proliferate and invade, contributing to tumor progression.

On the other side, at the advancement stages, autophagy not only provides energy and metabolic substrates for cancer cells, but also creates a microenvironment conducive to tumor growth [79]. It facilitates cancer progression by modulating antigen processing and presentation, suppressing T cell activation, and reducing the likelihood of recognition by natural killer (NK) cells, thereby enabling tumor cells to evade immune surveillance [80]. In addition, certain types of autophagy exert protective effects on tumor cells. For example, mitochondrial autophagy can help cancer cells avoid death by inhibiting mitochondrial outer membrane permeabilization, while endoplasmic reticulum autophagy (ER‐phagy) can enhance the endoplasmic reticulum's capacity to cope with stressful environments and benefit the cancer cells [81]. The increasing ability of the endoplasmic reticulum to handle stress is beneficial for cancer cell survival [82].

In addition, autophagy is involved in the induction of chemoresistance, which enhances cancer cell resistance to chemotherapeutic drugs while maintaining genetic stability [83]. This process is often mediated by various factors including AMP‐activated protein kinase (AMPK), AKT serine/threonine kinase (AKT), autophagy‐related proteins (ATGs), non‐coding RNAs, etc., whose regulation has been linked to cisplatin resistance in cancer [84]. Moreover, the prompt elimination of damaged organelles and proteins reduces the cytotoxicity of chemotherapeutic drugs, which results in acquired resistance [85].

To sum up, autophagy has two sides that cause an overall inhibitory effect in the early stages of cancer and more of a facilitatory effect in the advanced stages [86].

### 3.3 Molecular Regulation of Autophagy in Ovarian Cancer

**FIGURE 2:**
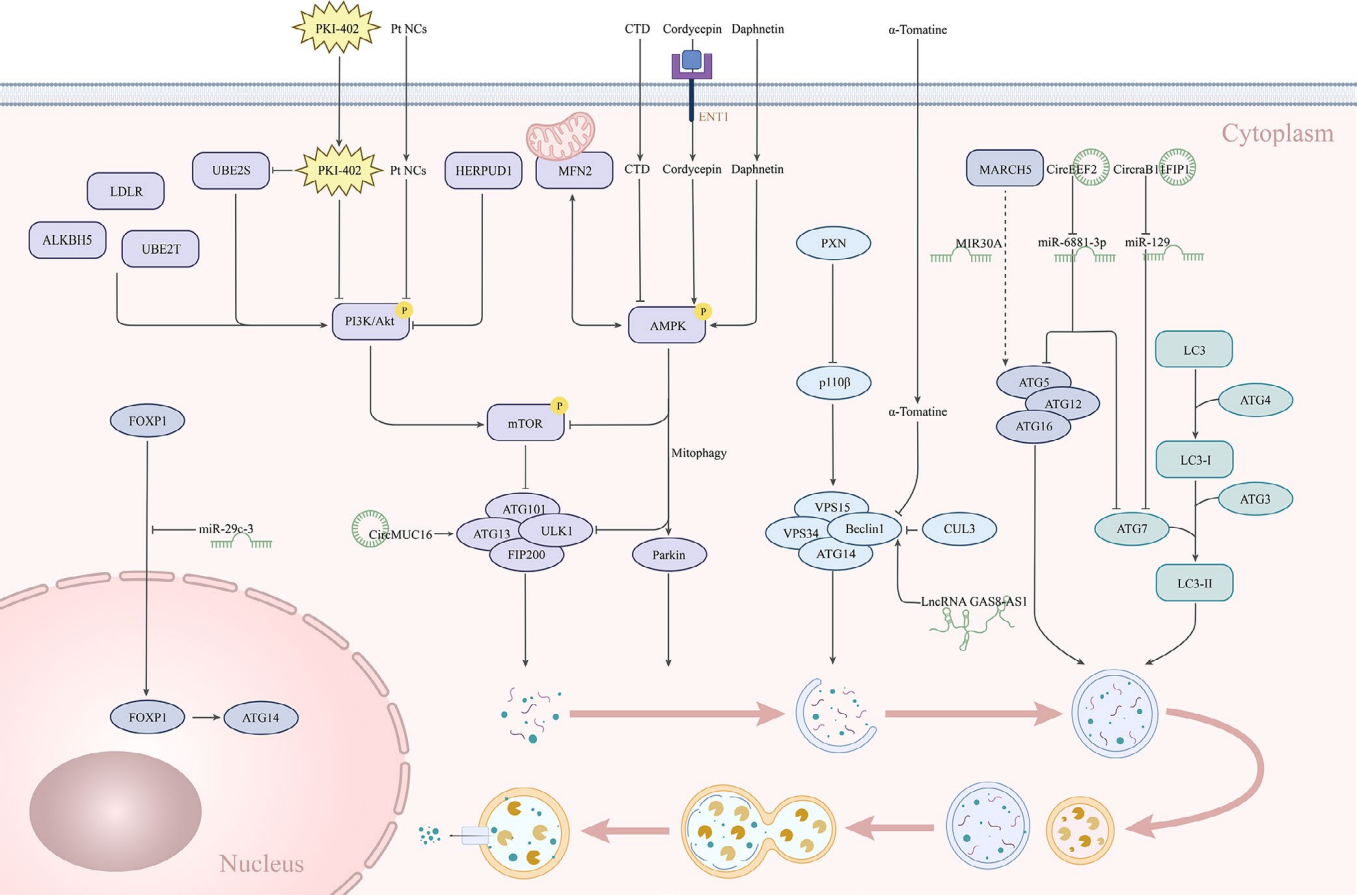
Specific targets of autophagy regulation in ovarian cancer.

Numerous signaling pathways and substances precisely regulate autophagy in ovarian cancer, ensuring its initiation, execution, and termination are carried out normally (Figure 2).

#### 3.3.1 Autophagy Activation

Several signaling molecules and pathways have been identified to positively regulate autophagy in ovarian cancer, which may either suppress tumor growth or paradoxically support cancer cell survival. Phosphatidylinositol 3‐kinase, or class IA PI3K, is an upstream regulator of several important autophagy‐related pathways. By controlling the formation of the PI3K complex, the catalytic subunit of class IA PI3K, p110 β, positively regulates autophagy via the p110β/Vps34/Beclin1 pathway [87, 88]. Additionally, Wu et al. discovered that Paxillin (PXN), which is significantly overexpressed in ovarian tissues, promotes ovarian cancer progression by modulating autophagy through this pathway [89]. Beclin1, a core scaffold protein in the PI3K complex, is a pivotal component of autophagy initiation. Long non‐coding RNA (LncRNA) GAS8‐AS1 activates autophagy by binding to Beclin1 and inhibits ovarian cancer progression, while α‐tomatine, a steroidal alkaloid extract, suppresses autophagy‐induced apoptosis by modulating Beclin1 activity [90, 91]. By inhibiting ubiquitination and Beclin1 degradation, suppression of cullin 3 (CUL3) activity enhances autophagy and inhibits the growth of ovarian cancer cells [92]. Furthermore, AMPK induces autophagy by functioning as an upstream regulator of mechanistic target of rapamycin (mTOR). Mitofusin 2 (MFN2), a mitochondrial fusion protein, activates AMPK to suppress mTOR and thereby triggers autophagy, ultimately inhibiting ovarian cancer cell growth [93]. Compounds such as cordycepin, which enters cells via nucleoside transporter protein Equilibrative nucleoside transporter 1 (ENT1), and daphnetin induce autophagy via the AMPK/mTOR axis, leading to cell death or cytoprotection in ovarian cancer cells [94, 95].

#### 3.3.2 Autophagy Suppression

In contrast, several upstream regulators and oncogenic pathways have been found to suppress autophagy, thereby contributing to ovarian cancer progression. The PI3K/AKT/mTOR signaling pathway is the most well‐established negative regulator of autophagy [96, 97]. ALKBH5, an N6‐methyladenosine (m6A) eraser protein, activates this pathway and suppresses autophagy, which facilitates ovarian cancer development [98]. Ubiquitin‐conjugating enzyme E2T (UBE2T) negatively regulates autophagy by maintaining the activity of this pathway, the level of which is negatively correlated with the prognosis of ovarian cancer patients [99]. Homocysteine‐inducible endoplasmic reticulum protein with ubiquitin‐like domain 1 (HERPUD1), however, acts in an opposite manner by inhibiting the PI3K/AKT/mTOR pathway and thus activating autophagy, which unexpectedly promotes ovarian cancer cell proliferation [100]. In addition, under energy deprivation, AMPK phosphorylation inhibits ULK1 activation and autophagy occurrence [101]. It can also directly inhibit mitochondrial autophagy in functionally normal mitochondria by triggering the sequestration of ULK1. However, when mitochondrial function is impaired, AMPK can directly phosphorylate Parkin to activate autophagy. This mechanism is essential for maintaining cellular energy balance and metabolic homeostasis [102].

#### 3.3.3 Regulation of ATGs

ATGs are central to the formation and maturation of autophagosomes. Circular RNA MUC16 (CircMUC16) promotes autophagy by directly binding to and upregulating ATG13, while the membrane‐associated RING‐CH5 proteins (MARCH5) regulate autophagy by competing with ATG5 and Suppressor of Mothers Against Decapentaplegic protein 2 (SMAD2) for microRNA 30A(MIR30A) binding [103, 104]. CircEEF2 directly binds to and inhibits the expression of microRNA 6881‐3p (miR‐6881‐3p) so that it indirectly upregulates ATG5 and ATG7 expression to induce autophagy, promoting ovarian cancer cell proliferation and invasion [105]. By downregulating miR‐129, CircRAB11FIP1 upregulates ATG7 and ATG14 to drive autophagy and accelerate tumor progression [106]. Forkhead box protein P1 (FOXP1) is another key transcription factor that binds directly to the ATG14 promoter to induce its expression. However, overexpression of miR‐29c‐3p reduces FOXP1 nuclear translocation, downregulates ATG14, and enhances platinum resistance by suppressing autophagy [107].

#### 3.3.4 Autophagy and Chemoresistance

Autophagy is closely implicated in the development of drug resistance in ovarian cancer. Not only can autophagy serve as a survival mechanism that helps cancer cells escape chemotherapy‐induced stress, but its inhibition can also sensitize cells to chemotherapeutic agents. Ubiquitin‐conjugating enzyme E2S (UBE2S) promotes cisplatin resistance by activating the PI3K/AKT/mTOR pathway and suppressing autophagy [108]. Similarly, when the low‐density lipoprotein receptor (LDLR) is downregulated or knocked down, this pathway is activated so that autophagy as well as autophagy‐mediated drug resistance are inhibited, especially in drug‐resistant ovarian cancer [109]. In contrast, PKI‐402, a dual PI3K/mTOR inhibitor, enhances autophagic flux and restores cisplatin sensitivity [110]. Additionally, the chemotherapeutic drug Pt NCs inhibited the expression of this pathway and UBE2S to activate autophagy in cisplatin‐resistant ovarian cancer cells [111]. By blocking the autophagosome‐lysosome fusion pathway, costunolide (CTD) sensitizes cells to cisplatin, disrupting autophagic flux [112].

These findings indicate that the impact of autophagy activation or suppression on tumor progression is context‐dependent, influenced by both the stage of cancer and the molecular environment. Given such roles of autophagy in ovarian cancer progression and drug resistance, targeting autophagy represents a promising therapeutic strategy. Modulation of autophagy via key signaling nodes—such as PI3K/AKT/mTOR and AMPK/mTOR pathways—could offer novel approaches to overcome chemoresistance and improve treatment efficacy. This also opens new avenues for natural compounds like Ginsenosides. These bioactive molecules, derived from ginseng, have shown the ability to regulate autophagy through the same pathways. Their multitarget effects and low toxicity make them attractive candidates for the development of novel small‐molecule drugs that synergize with existing therapies to combat ovarian cancer.

#### 3.3.5 Roles of Autophagy‐Related miRNAs in Ovarian Cancer

Among the ~3000 miRNAs reported to date, hundreds have been implicated as direct regulators of autophagy. Genes encoding key proteins that function at distinct stages of the autophagy pathway are frequent miRNA targets, enabling miRNAs to form a multilayer regulatory network that spans autophagy initiation, elongation, and maturation [113]. Accumulating evidence highlights the importance of miRNA‐mediated autophagy regulation in ovarian cancer and links this axis to tumor behavior and therapeutic responses [114]. Notably, miRNA‐driven modulation of autophagy has been reported to influence the radiosensitivity of ovarian cancer cells [115].

At the clinical level, one study analyzed 31 ovarian cancer patients who underwent miRNA sequencing and validated the findings across multiple independent datasets. The selected miRNA signature showed high diagnostic accuracy for stage I high‐grade serous ovarian cancer (AUC = 0.99), supporting its potential value for early detection [116]. Consistently, miRNA‐4478 is maintained at low levels in ovarian cancer, and its expression decreases over time in irradiated ovarian cancer cells. This decline is associated with poor prognosis, suggesting a possible role in radiotherapy‐related autophagy responses [115]. Beyond tumor cells, autophagy is also essential for endothelial function and angiogenesis. It has been shown that lncRNA ANRIL can interfere with autophagy via miRNA‐99a and miRNA‐449a, thereby promoting angiogenesis [117]. Regarding tumor progression, certain miRNAs can affect migration and metastasis by negatively regulating autophagy. For example, Song et al. reported that miRNA‐219‐5p targets HMGA2 to suppress Wnt/β‐catenin signaling and autophagy, which attenuates cisplatin resistance and significantly inhibits proliferation and migration of ovarian cancer cells [118]. Conversely, in specific molecular contexts, miRNA‐induced autophagy activation may also contribute to the regulation of migration and metastasis, further underscoring the context‐dependent nature of the miRNA–autophagy axis in ovarian cancer [119].

#### 3.3.6 Additional Autophagy‐Related Signaling Axes Shaping Ovarian Cancer Progression

Beyond the canonical autophagy regulatory modules summarized above, a broader signaling landscape also critically shapes autophagy‐dependent phenotypes in ovarian cancer. Accumulating evidence indicates that modulation of key components within the PI3K/AKT/mTOR pathway can directly influence ovarian cancer cell proliferation, migration, and chemoresistance. For example, HERPUD1, a protein implicated in ubiquitination and degradation of misfolded proteins, suppresses PI3K/AKT/mTOR signaling, thereby inducing autophagy and restraining epithelial–mesenchymal transition (EMT) [100]. In contrast, HIF‐1α, a central regulator of cellular hypoxia responses, can activate PI3K/AKT/mTOR signaling to downregulate autophagy and promote ovarian cancer cell proliferation [120]. Autophagy regulation in ovarian cancer is also intertwined with Ras/ERK signaling. ARHI, an imprinted tumor suppressor gene, inhibits the Ras/ERK pathway, which reduces FOXO3a phosphorylation and promotes its nuclear retention. This transcriptional shift upregulates ATG4 and LC3‐I—both essential for autophagosome maturation—as well as Rab7, a key mediator required for autophagosome–lysosome fusion. Collectively, these changes limit ovarian cancer cell growth [121]. In addition to oncogenic signaling and hypoxic adaptation, stress‐responsive pathways can be pharmacologically leveraged to rewire autophagy and overcome resistance. Triptolide (TPL) increases intracellular ROS, suppresses JAK2/STAT3 signaling, and downregulates Mcl‐1. The resulting attenuation of Mcl‐1–mediated Beclin‐1 inhibition ultimately promotes cell death in chemoresistant ovarian cancer cell lines [122]. Consistently, inhibition of the Nrf2/HO‐1 axis enhances ROS‐dependent apoptosis and autophagy, further suppressing ovarian cancer cell proliferation and migration [123].

The impact of autophagy activation or inhibition on tumor progression is highly context‐dependent, shaped jointly by tumor stage and the surrounding molecular milieu. Given the roles of autophagy in ovarian cancer progression and drug resistance, targeting autophagy holds therapeutic potential. Modulating autophagy through key nodes such as the PI3K/AKT/mTOR and AMPK/mTOR pathways may offer new strategies to overcome chemoresistance and improve treatment efficacy. This also opens a promising research avenue for natural compounds such as ginsenosides, which can regulate autophagy via the same pathways and, with their multitarget actions and relatively low toxicity, may serve as novel small‐molecule candidates that synergize with existing therapies to combat ovarian cancer.

## 4 Ginsenosides in Cancer: From Structure to Therapeutic Mechanisms

Ginseng is a traditional Chinese medicine that has been used for centuries as a well‐known herb in both traditional and modern medicine. It exhibits a wide range of functions including boosting immunity, relieving fatigue, improving memory, and antioxidant [124]. According to traditional Chinese medicine formulas, ginseng can be used for numerous gynecological problems, such as irregular menstruation, metrorrhagia (abnormal uterine bleeding), and deficiency of qi and blood [125]. Ginsenosides, the primary pharmacologically active ingredients in ginseng, have shown promising anticancer effects across multiple cancer types and can regulate autophagy in multiple ways. They have been confirmed to inhibit tumor growth, promote apoptosis, reverse drug resistance, and enhance cell death in cancers such as cervical, liver, breast, colorectal, gastric, and lung cancer through autophagy modulation [126, 127, 128, 129, 130, 131, 132, 133, 134].

### 4.1 Structure and Anticancer Functions of Ginsenosides

Ginsenosides are tetracyclic glycosylated triterpenoid saponins consisting of thirty carbon atoms, containing a tetracyclic steroidal nucleus and a variety of sugar moieties. More than two hundred isoforms have been isolated and identified to date [135]. Based on their chemical structure, they can be divided into two groups: oleanolic acid and dammarane types. The majority of dammarane types possess a tetracyclic skeleton structure and an extra sugar moiety. These types can be further divided into protopanaxadiol (PPD) and protopanaxatriol (PPT). The primary distinction between the two is the presence of an α‐configurational hydroxyl group at PPT's C‐6 position; PPD contains Rb1, Rb2, Rb1, Rb2, Rb3, Rc, Rd., Rg3, and Rh2, etc., whereas PPT contains Re, Rg1, Rg2, and Rh1, etc. Oleanolic acid serves as the mother nucleus for oleanolic acid‐type saponins, which have a pentacyclic triterpene skeleton structure and common constituents such as Ro, Rh3, and Ri. The structural diversity of ginsenosides is closely related to their biological activities. The hydroxyl group at the C‐6 position of PPT makes it easier to be dehydrated and transformed, and the carboxyl group at the C‐28 position of oleanolic acid‐type saponins enhances their water solubility [10, 136]. It has been shown that the number of sugar residues in ginsenoside molecules is inversely proportional to their antitumor activity, but the specific molecular mechanism underlying this relationship remains unclear [137].

The main mechanisms by which ginsenosides exert anticancer effects include apoptosis induction, cell cycle arrest and modulation of the immune system. Apoptosis is an important target for cancer treatment. Rg5 induces apoptosis and cell cycle arrest in gastric cancer by upregulating intracellular ROS levels, activating the MAPK pathway, and lowering the mitochondrial membrane potential [129]. It also induces apoptosis and cell cycle arrest in breast cancer cells by direct inhibition of the anti‐apoptotic protein B‐cell lymphoma 2 (Bcl‐2), lowering the phosphorylation levels of PI3K and Akt, and blocking the PI3K/Akt signaling pathway [128]. Rh2 targets and inhibits the mitochondrial electron transport chain complex in cervical cancer HeLa cells, which induces mitochondrial ROS accumulation and promotes apoptosis [138]. It also upregulates the expression of microRNA‐3614‐3p by inhibiting lncRNA CFAP20DC‐AS1 in breast cancer, which in turn inhibits oncogenes Bobby Sox homolog (BBX) and tumor necrosis factor alpha‐induced protein 3 (TNFAIP3) axis to induce apoptosis [139].

Dysregulation of cell cycle is one of the core mechanisms of cancer development, and the loss of control of key nodes (e.g., G1/S, G2/M) leads to aberrant cell proliferation and genomic instability. Rg5 inhibits proliferation of gastric cancer cells and non–small‐cell lung cancer (NSCLC) cells by regulating cell cycle regulatory proteins inducing the blockage of the G2/M phase [127, 129]. Rh1 inhibits cell proliferation by upregulating mitochondrial ROS levels in gastric cancer and induces endoplasmic reticulum stress to block breast cancer cells in the G1/S phase, thereby inhibiting growth [140].

In addition, ginsenosides modulate the immune system. Rh2 stimulates T‐cell infiltration, which changes the immune microenvironment and enhances the immune response by triggering immune‐activating factors and inhibiting immune‐suppressing factors. It also encourages CD8^+^ T‐cell and NK‐cell activation and increases sensitivity to immunotherapies and chemotherapy [141]. In breast cancer, Rh2 enhances the immunosurveillance capacity and killing power of NK cells by directly binding to and inhibiting the expression of endoplasmic reticulum protein 5 (ERp5), inhibiting the growth and metastasis of cancer cells [142].

The process known as the EMT causes epithelial cells to become less polar and take on characteristics of mesenchymal cells, which encourages the migration and invasion of cancer cells. In gastric cancer, Rh2 and CK enhance the chemosensitivity of gastric cancer cells by reversing EMT through inhibiting mesenchymal markers and promoting the expression of epithelial markers, and CK can also directly target and inhibit the phosphorylation of PI3K/Akt, which doubly blocks the activation of downstream EMT transcription factors [143, 144].

**FIGURE 3:**
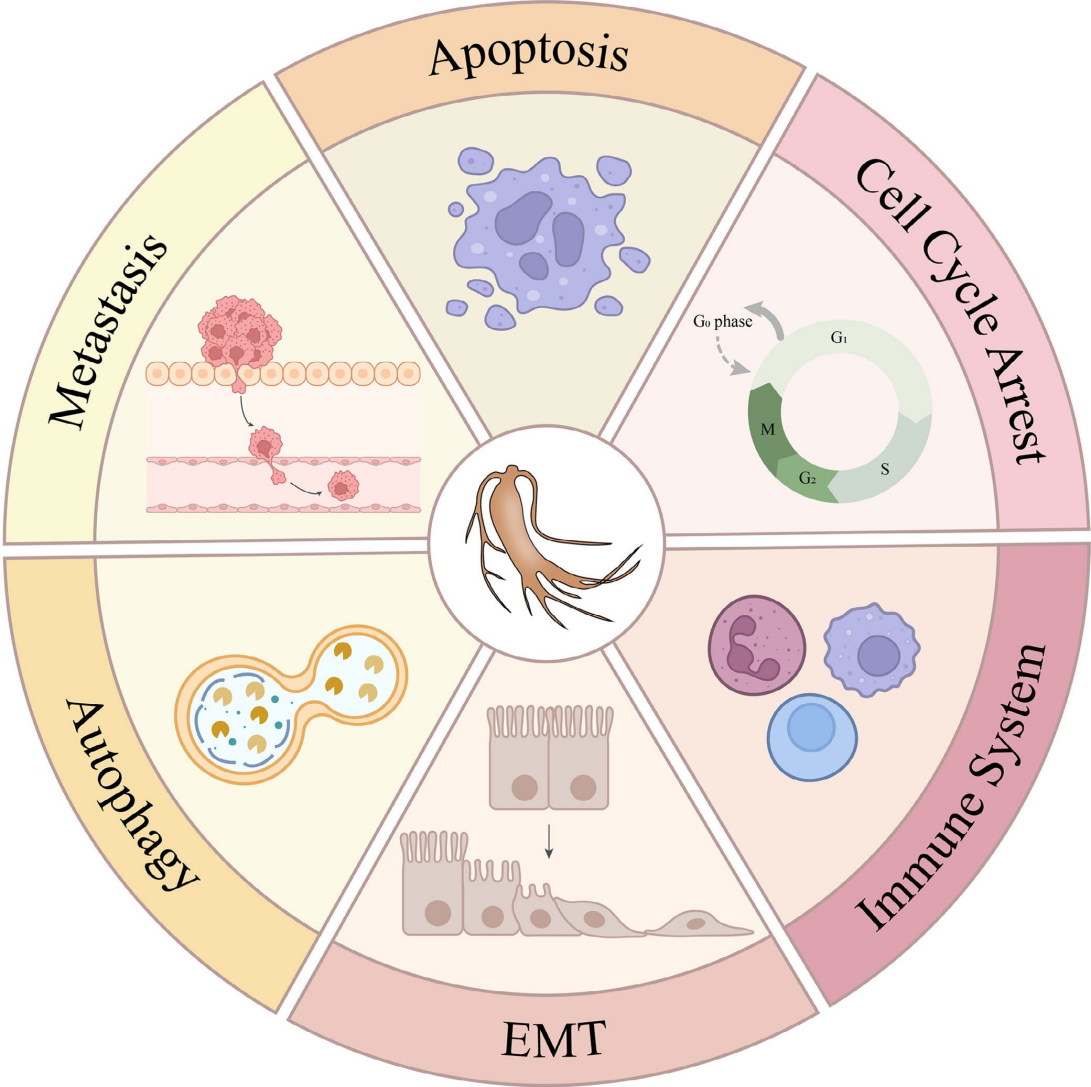
Mechanisms by which ginsenosides act against tumors. By encouraging apoptosis, causing cell cycle arrest, influencing the immune system, modulating the EMT process, controlling autophagy, and preventing invasion, ginsenosides slow the spread of cancer development.

Building on these diverse anticancer mechanisms, ginsenosides also play a crucial role as modulators of autophagy in cancer(Figure 3).

### 4.2 Ginsenosides as Autophagy Modulators in Cancer

By acting at multiple stages of the autophagic process, ginsenosides modulate autophagy and inhibit cancer progression. As a crucial regulator, p53 promotes the transcription of autophagy‐related genes and initiates the autophagy cascade [145]. Rh4 dramatically increases intracellular ROS levels, which subsequently activate the ROS/JNK/p53 signaling pathway. This activation leads to enhanced autophagosome formation, as indicated by increased LC3‐II levels and decreased p62, indicating successful autophagosome‐lysosome fusion and normal substrate degradation. Autophagy activation then promotes ferroptosis, inhibiting colorectal cancer cell proliferation [146]. Similarly, Rk1 activates AMPK to modulate the AMPK/mTOR pathway, resulting in elevated LC3‐II and reduced p62 levels in hepatocellular carcinoma cells, thereby promoting autophagy and apoptosis [147]. Rh2 induces conversion of LC3‐I to LC3‐II through inhibition of the AMPK/mTOR signaling pathway in cervical carcinoma cells, promoting protective autophagy and apoptosis [130]. Furthermore, by reducing phosphorylation levels of PI3K, Akt, and mTOR, Rg5 inhibited the PI3K/Akt pathway, promoting the fusion of autophagic vesicles with lysosomes, increasing autophagic lysosomes and apoptosis, and inhibiting the proliferation of breast cancer cells [128]. In lung cancer cells, Rg3 elevates both LC3‐II and p62 levels, suggesting inhibition of autophagic flux through blockade of autophagosome degradation, which overcomes ectinib resistance and improves its therapeutic efficacy when combined with ectinib [148].

In addition, ginsenoside fermentation products (FTGs) and derivatives also modulate autophagy in cancer, thereby inhibiting tumor progression. FTGs, with large amounts of rare ginsenosides, directly or indirectly induce autophagy in colorectal cancer through activation of the AMPK/mTOR pathway or inhibition of the JAK2/STAT3 pathway [149]. 2‐deoxy‐Rh2, a novel 20(s)‐ginsenoside Rh2 derivative, promotes autophagy in breast cancer cells via the AMPK/mTOR pathway, up‐regulating LC3‐II and downregulating p62 [134].

Autophagy and apoptosis interact with each other and are inseparable in cancer progression. Autophagy can inhibit or promote apoptosis by targeting mitochondria or apoptosis‐related proteins, while apoptosis regulates autophagy via caspase‐mediated cleavage or apoptotic body clearance. Key regulators such as Beclin1 and p53 modulate both autophagy and apoptosis, and disruption of the dynamic balance between these two processes is considered a critical factor in tumorigenesis [150]. Ginsenosides help to balance autophagy and apoptosis while controlling autophagy. PPD, a major ginsenoside subtype, induces autophagy in gastric cancer cells by inhibiting Src phosphorylation, thereby suppressing Akt/mTOR signaling, increasing the LC3‐II/LC3‐I ratio, and upregulating ATG5, ATG7, and Beclin1. It also promotes apoptosis by activating caspases and increasing apoptotic cell populations, mainly via Src inhibition [151]. In cervical carcinoma cells, Compound K (CK), a major terminal metabolite of PPD, induced autophagy by upregulating Beclin1 and Atg5, disrupting mitochondrial membrane potential, and triggering endoplasmic reticulum stress. It also increases apoptosis‐related markers such as caspases and Bak. Moreover, treatment with the autophagy inhibitor 3‐MA significantly enhanced apoptosis, while the apoptosis inhibitor Z‐LEHD‐FMK elevated autophagy‐related protein levels, further suggesting that CK likely regulates both autophagy and apoptosis in a coordinated manner [131]. Rk3 induced autophagy in hepatocellular carcinoma cells by suppressing the PI3K/AKT signaling pathway. Importantly, both in vitro and in vivo experiments with Rk3 treatment showed increased levels of apoptosis, implying its potential role in regulating both autophagy and apoptosis [152]. Although the precise mechanism and the intricate regulatory relationship between autophagy and apoptosis are still unknown, ginsenosides' ability to modulate the balance between the two provides promising directions for future studies.

It should be noted that autophagy comprises three major forms: macroautophagy, microautophagy, and chaperone‐mediated autophagy [153]. Currently, research on ginsenoside‐mediated autophagy in disease intervention mainly focuses on macroautophagy, with relatively fewer studies on the other forms of autophagy [154]. Studies have shown that ginsenosides can enhance autophagy in neuronal cells, characterized by increased LC3‐II and Beclin‐1 levels and decreased p62 levels [155]. In non‐small cell lung cancer (NSCLC), Rh2 and Rg3 induce autophagic cell death through an ER stress–autophagy axis and modulate choline–phosphatidylcholine metabolism [156]. Moreover, certain ginsenosides (e.g., Rk3 and Rg5) and their optimized extracts can induce autophagy in various cancer types, including esophageal, breast, and lung cancers, by inhibiting the PI3K/AKT/mTOR signaling pathway or activating ULK1 phosphorylation, thereby suppressing tumor growth and proliferation [154]. Notably, Rg3 has also been reported to enhance PI3K and AKT phosphorylation in non‐malignant contexts, preventing excessive autophagy activation and thereby limiting the progression of hepatic fibrosis. This highlights the context‐dependent nature of ginsenoside‐mediated autophagy regulation [157]. Due to low drug concentrations upon oral administration, which limit its effective delivery to ovarian cancer (OC) sites, ginsenoside Rg3 is being delivered directly to the ovarian tumor surface via microneedles (MNs) made from methacrylated gelatin, which have good biocompatibility and drug‐loading efficiency, thus exerting anti‐tumor effects [158]. Rg3 regulates multiple signaling pathways, including phosphoinositide 3‐kinase, epidermal growth factor receptor, mitogen‐activated protein kinase, p53, nuclear factor‐kappa B, and reactive oxygen species, exhibiting anti‐cancer activity both in vitro and in vivo [159]. Additionally, Rg3 inhibits starvation‐induced autophagic flux in HeLa ATCC and CCL‐2 cells, significantly increasing LC3‐II and p62 protein levels [160].

In conclusion, ginsenosides exert anticancer effects by modulating autophagy through multiple signaling pathways and key regulatory proteins, thereby restoring autophagic balance and suppressing tumor progression. Further investigation into these mechanisms will advance the development of ginsenoside‐based therapies targeting autophagy in cancer treatment.

### 4.3 Antitumor Mechanisms of Ginsenosides in Ovarian Cancer

Various isoforms of ginsenosides have shown significant potential in inhibiting the progression of ovarian cancer, exerting antitumor effects through diverse but interconnected mechanisms. Ginsenosides inhibit tumor growth and metastasis, promote cell apoptosis, and thereby suppress tumor cells. Additionally, ginsenosides enhance sensitivity to traditional chemotherapy agents in ovarian cancer [161]. Currently, clinical evidence focusing specifically on “ginsenosides in ovarian cancer” is relatively scarce, with most studies centered on Rg3 (Ginsenoside A) as an adjuvant to chemotherapy. However, preclinical research in this area is more comprehensive.

First, several ginsenosides disrupt cell cycle progression and inhibit proliferation. Rg3, a well‐characterized PPD‐type ginsenoside, downregulates KIF20A by inhibiting NF‐κB signaling, thereby promoting CDC25A proteasomal degradation and inducing G1‐phase arrest [162]. Rk1 similarly induces G1‐phase arrest in SKOV3 cells, accompanied by ROS accumulation, mitochondrial dysfunction, and caspase‐mediated apoptosis [163]. Rg5 impairs tumor cell viability and invasiveness via fibroblast growth factor‐8b (FGF8b) downregulation and G1 arrest [164].

Second, ginsenosides regulate epigenetic modifications to restore tumor suppressor function. Rg3 reverses abnormal DNA hypermethylation, restoring the expression of tumor suppressor genes p53 and p16, thereby inhibiting ovarian cancer cell proliferation, invasion, and metastasis [165]. Additionally, ginsenoside 20(S)‐Rg3 attenuates the Warburg effect—an important hallmark of cancer metabolism that promotes tumor progression—by downregulating the DNA methyltransferase DNMT3A‐mediated methylation and upregulating miR‐519a‐5p, thereby suppressing HIF‐1α signaling and inhibiting tumor growth [166, 167]. Other epigenetic mechanisms include suppression of oncogenic non‐coding RNAs such as miR‐4425 and lncRNA H19, reducing Farnesyl‐Diphosphate Farnesyltransferase 1 (FDFT1) expression and ovarian cancer cell migration and proliferation [168, 169]. Additionally, 20(S)‐Rg3 exerts its antitumor effects by reprogramming cholesterol metabolism through the HIF‐1α/SQLE/FDFT1 pathway [170]. For instance, 20(S)‐Rg3 has been shown to enhance autophagic flux in the SKOV3 ovarian cancer cell line, evidenced by increased LC3‐II levels and enhanced autophagosome‐lysosome fusion. Notably, the inhibition of autophagy reverses part of its antitumor effects [171].

Third, ginsenosides modulate the tumor immune microenvironment and stemness. Rh2 enhances natural killer (NK) cell activity, contributing to an indirect antitumor response [141]. Rb1 and its metabolite Compound K suppress the self‐renewal of ovarian cancer stem cells and increase chemosensitivity by downregulating the Wnt/β‐catenin pathway and inhibiting epithelial–mesenchymal transition (EMT) [172].

Extending far beyond the classical hallmarks of ginsenoside antitumor activity—cell‐cycle arrest, epigenetic reprogramming, metabolic rewiring, immunomodulation, and stemness suppression—an accumulating body of work now positions autophagy as a nexus through which these triterpenoid saponins exert cytotoxic and chemosensitizing actions in ovarian cancer.

In SKOV3 cells, 20(S)‐Rg3 enhances autophagic flux, evidenced by elevated LC3‐II levels and increased autophagosome‐lysosome fusion in mRFP‐GFP‐LC3 assays. This effect, linked to upregulated ATG5 and ATG7 expression, correlates with reduced migration and invasion. Notably, autophagy inhibition with chloroquine reverses these antitumor effects, confirming autophagy as a key mediator [171]. Beyond autophagy, 20(S)‐Rg3 induces apoptosis by suppressing the PI3K/Akt/mTOR axis in HO‐8910 cells. This inhibition downregulates Bcl‐2 and IAPs while upregulating Bax, leading to caspase activation and apoptosis. Given mTOR's dual role in autophagy and apoptosis, 20(S)‐Rg3 likely coordinates tumor suppression through both pathways [173]. Similarly, the rare ginsenoside Rg6 reverses cisplatin resistance by targeting GRB2, thereby inhibiting the GRB2–ERK1/2–mTOR cascade. This relieves mTOR‐mediated ULK1 repression, enhances Beclin‐1/VPS34 interaction, and upregulates ATG5, ATG7, and LC3‐II, promoting autophagosome initiation. Concurrent ERK1/2 suppression further disrupts mitogenic signaling, while autophagy induction sensitizes ovarian cancer cells to cisplatin [174]. Additionally, ginsenoside Rg3 suppresses the transcription of KIF20A and promotes the proteasomal degradation of CDC25A in epithelial ovarian cancer, exerting its antitumor effects [175].

In summary, ginsenosides regulate key molecular pathways to modulate autophagy, suppress ovarian cancer progression, and overcome chemoresistance. They act through various mechanisms, with mTOR serving as a central regulatory hub, positioning autophagy as a crucial therapeutic target. Ginsenosides' structural diversity (PPD/PPT/oleanolic acid types and sugar moiety variations) allows multi‐target regulation of autophagy via pathways such as ROS/JNK/p53, AMPK/mTOR, PI3K/Akt/mTOR, and JAK2/STAT3. Depending on context, they can enhance autophagic flux to trigger apoptosis/ferroptosis or inhibit autophagosome degradation to overcome drug resistance. Additionally, ginsenosides influence cell‐cycle progression, epigenetic modifications, metabolic reprogramming, and the immune microenvironment, further enhancing their antitumor effects. Overall, these findings suggest that ginsenosides exert integrated effects in ovarian cancer by linking autophagy, apoptosis, and resistance reversal, leading to suppressed tumor proliferation, reduced invasion, and enhanced chemosensitivity. These multifaceted pathways by which ginsenosides inhibit cancer progression across various diseases are summarized in Table 2. However, current research remains in the early stages, and further studies are needed to fully understand the complex regulatory networks and optimize ginsenoside‐based therapies for precise modulation of autophagy in ovarian cancer treatment.

**TABLE 2:** Multiple pathways of ginsenosides to inhibit cancer progression in different diseases.

Drug	Subtype	Disease	Function	Pathway/targets	Mechanism	References
Rg3	PPD	Non‐small cell lung cancer	Inhibition of autophagy	Autophagic degradation phase	Upregulation of both LC3‐II and p62 inhibits autophagosomal degradation and reverses chemoresistance.	[148]
Rg3	PPD	Ovarian cancer	Regulation of the cell cycle	Key cell cycle regulators	Inhibition of the NF‐κB pathway downregulates KIF20A, promotes proteasomal degradation of CDC25A and induces G1 phase blockade.	[162]
Rg3	PPD	Ovarian cancer	Epigenetic modification	DNA methylation, non‐coding RNA	Inhibition of promoter methylation promotes transcription and translation of oncogenes p53 and p16 and upregulates their expression; reduction of DNMT3A‐mediated DNA methylation, upregulation of miR‐519a‐5p expression, and inhibition of the HIF‐1α‐mediated Warburg effect; downregulation of miR‐4425 expression, and deregulation of its inhibition of the oncogene FDFT1; downregulation of lncRNA H19 expression.	[165, 167, 168, 169]
Rg3	PPD	Ovarian cancer	Promoting autophagy	—	LC3 II, ATG5 and ATG7 were upregulated, autophagy flux was increased and migration and invasion of ovarian cancer cells were significantly reduced.	[171]
Rg3	PPD	Ovarian cancer	Induction of apoptosis	Akt/mTOR signaling pathway	Phosphorylation levels of Akt and its downstream molecule mTOR were significantly reduced.	[173]
Rg5	PPD	Gastric cancer	Induction of apoptosis, cell cycle regulation	ROS, MAPK pathway	Activation of the ROS‐mediated MAPK pathway blocks cells in G2/M phase.	[129]
Rg5	PPD	Breast cancer	Induction of apoptosis, promotion of autophagy	Apoptosis‐related protein, PI3K/Akt pathway	Directly inhibits the anti‐apoptotic protein Bcl‐2, decreases the phosphorylation level of PI3K and Akt; promotes the fusion of autophagic vesicles with lysosomes and increases the number of autophagic lysosomes.	[128]
Rg5	PPD	Non–small‐cell lung cancer	Regulation of the cell cycle	PI3K/Akt/mTOR pathway	Inhibitory pathways modulate cell cycle protein expression to induce G2/M phase blockade.	[127]
Rg5	PPD	Ovarian cancer	Inhibition of proliferation, migration and invasion	Fibroblast growth factor‐8b (FGF8b)	Downregulation of FGF8b expression inhibits ovarian cancer cell viability.	[164]
Rg6	PPT	Ovarian cancer	Promoting autophagy	Growth factor receptor binding protein 2 (GRB2)	Directly binds to GRB2, downregulates mTOR, and promotes autophagy initiation and autophagosome membrane formation; the LC3‐II/LC3‐I ratio is significantly elevated, and the increase in autophagic flux significantly inhibits the proliferation of ovarian cancer cells.	[174]
Rh1	PPT	Breast cancer	Regulation of the cell cycle	Mitochondrial pathway, endoplasmic reticulum stress	Upregulates mitochondrial reactive oxygen species levels and induces endoplasmic reticulum stress, induces G1/S phase blockade and inhibits growth.	[140]
Rh2	PPD	Cervical cancer	Induction of apoptosis, promotion of autophagy	Mitochondrial pathway, AMPK/mTOR pathway	Inhibits mitochondrial electron transport chain complexes and induces ROS accumulation; promotes LC3B I to LC3B II conversion.	[130, 138]
Rh2	PPD	Breast cancer	Induction of apoptosis, modulation of immunity	Epigenetic modifications, immune microenvironment	Inhibition of lncRNA CFAP20DC‐AS1 up‐regulates microRNA‐3614‐3p and suppresses oncogenic genes BBX and TNFAIP3 axis; inhibition of ERp5 enhances the immune surveillance ability and killing power of NK cells.	[139, 142]
Rh2	PPD	Gastric cancer	Modulation of immunity	EMT	Downregulation of mesenchymal markers, upregulation of epithelial markers, and the reversal of EMT process enhances chemosensitivity.	[141]
Rh2	PPD	Ovarian cancer	Modulation of immunity	Immune microenvironment	Enhancement of NK cell killing and activity inhibits ovarian cancer cell proliferation.	[141]
2‐deoxy‐Rh2	Ginsenoside derivatives	Breast cancer	Promoting autophagy	AMPK/mTOR pathway	LC3‐II is upregulated and p62 is downregulated to promote autophagic vesicle formation.	[134]
Rh4	PPD	Colorectal cancer	Promotes autophagy, promotes iron death	ROS/JNK/p53 pathway	Increases intracellular reactive oxygen species content thereby activating the ROS/JNK/p53 pathway, up‐regulates LC3‐II, decreases p62, promotes autophagosome formation, and promotes iron death.	[146]
Rb1	PPD	Ovarian granulosa cell‐related diseases	Inhibition of apoptosis	Akt	Increasing the phosphorylation level of Akt at Ser473 site enhances its activity and inhibits oxidative stress‐induced apoptosis and damage in ovarian granulosa cells.	[176]
Rb1 and its metabolites	PPD	Ovarian cancer	Enhanced drug sensitivity	Wnt/β‐catenin pathway, EMT	Inhibition of self‐renewal of ovarian cancer stem cells and enhancement of cancer cell sensitivity to chemotherapeutic agents through inhibition of the Wnt/β‐catenin pathway and epithelial‐mesenchymal transition.	[172]
Rk1	PPD	Liver cancer	Promotes autophagy, induces apoptosis	AMPK/mTOR pathway	Activation of AMPK proteins regulates the AMPK/mTOR pathway with upregulation of LC3‐II and a decrease in p62.	[147]
Rk1	PPD	Ovarian cancer	Induction of apoptosis, cell cycle regulation	Mitochondrial pathway	Up‐regulation of ROS levels and decrease in mitochondrial membrane potential followed by activation of caspase signaling pathway induced apoptosis; induction of G1 phase arrest.	[163]
Rk3	PPT	Liver cancer	Promotes autophagy, induces apoptosis	PI3K/AKT signaling pathway	Decreased phosphorylation levels of PI3K and AKT inhibited the PI3K/AKT pathway to induce autophagy; in combination with apoptosis inhibitors resulting in decreased autophagy levels.	[152]
CK	PPD	Gastric cancer	Modulation of immunity	Suppression of EMT	Dual reversal of the EMT process through modulation of mesenchymal markers and direct targeting to inhibit PI3K/Akt phosphorylation enhances chemosensitivity.	[144]
CK	PPD	Cervical cancer	Promotes autophagy and inhibits apoptosis	Mitochondrial pathway, endoplasmic reticulum stress	Up‐regulates the expression of autophagy proteins such as Beclin1 and Atg5, inhibits mitochondrial membrane potential, and promotes endoplasmic reticulum stress; inhibits the increase in autophagy followed by apoptosis, and inhibits the increase in autophagy followed by apoptosis.	[131]
PPD	PPD	Gastric cancer	Promoting autophagy	Src/Akt/mTOR signaling pathway	Elevated LC3‐II/LC3‐I ratio and upregulation of ATG5, ATG7, Beclin1.	[151]
FTGs	Ginsenoside fermentation products	Colorectal cancer	Promoting autophagy	AMPK/mTOR pathway, JAK2/STAT3 pathway	Activation of AMPK/mTOR pathway or inhibition of JAK2/STAT3 pathway directly or indirectly induces autophagy.	[149]

## 5 Discussion and Perspective

Ovarian cancer therapy continues to face major challenges, largely driven by chemotherapy‐associated toxicities and the frequent emergence of drug resistance. As a therapeutic target, autophagy exhibits a canonical “double‐edged sword” behavior. In early‐stage disease or under specific stress conditions, autophagy may suppress tumorigenesis by maintaining cellular homeostasis and removing damaged components. In advanced disease, however, the same cytoprotective program can be exploited by cancer cells to adapt to hypoxia, nutrient deprivation, and therapeutic stress, thereby supporting tumor survival and facilitating acquired resistance. Importantly, the literature does not uniformly agree on whether autophagy is predominantly tumor‐suppressive or tumor‐promoting in ovarian cancer. Such discrepancies likely reflect differences in disease stage, molecular context, microenvironmental cues, and treatment modalities and timing. Therefore, future work should prioritize context‐aware study designs, incorporating dynamic measurements of autophagic flux and biomarker‐based stratification to avoid oversimplified, one‐directional interpretations.

Autophagy regulation in ovarian cancer is highly complex. It involves classical signaling axes such as PI3K/AKT/mTOR and AMPK/mTOR, as well as less‐explored but potentially more specific nodes including the p110β/Vps34/Beclin1 pathway, Beclin1 itself, and ATG proteins. Therapeutic outcomes may vary by the node and the stage of the autophagy process being targeted: inhibiting certain components can sensitize tumors to therapy, whereas activating or blocking different steps (initiation, membrane elongation, fusion, and degradation) may yield divergent biological consequences. Particularly in resistant settings, autophagy can function as a survival mechanism but may also contribute to cell‐death programs under defined conditions. Thus, translating autophagy modulation into actionable therapeutic strategies requires a stage‐ and flux‐informed framework rather than reliance on static markers alone.

Clinical translation is further constrained by pharmacokinetic and formulation barriers. Structural diversity implies isoform‐specific physicochemical properties, biological activities, and pharmacological effects, underscoring the need for systematic mapping of “isoform–pathway–response” relationships. Orally, limited intestinal absorption—particularly for lipophilic isoforms—together with extensive hepatic metabolism reduces systemic exposure [177], while multiple and variable in vivo biotransformation routes complicate pharmacokinetic evaluation [178]. Low solubility and poor intestinal permeability are major contributors to poor bioavailability and hinder clinical translation [179]. In addition, the inherent lipophilicity of several ginsenosides may limit selective accumulation in target tissues, thereby constraining therapeutic efficacy [180]. To mitigate these barriers, delivery approaches such as nanoparticle encapsulation, micellar formulations, and polymer–drug conjugates have been explored [179, 181]. These platforms aim to improve solubility and stability, prolong circulation, and enable controlled and/or targeted release; however, their in vivo consistency, scalability, long‐term safety, and clinical manufacturability still require further validation and standardization. From a biotransformation perspective, high–molecular‐weight ginsenosides often require deglycosylation by gut microbiota–derived enzymes to yield more absorbable low–molecular‐weight glycosides or aglycones [182]. After oral administration, ginsenosides are converted into multiple bioactive metabolites through enzymatic activities in the gastrointestinal tract and liver, with additional contributions from gastric conditions, digestion, and the intestinal microbiome; this conversion is critical for absorption and therapeutic effects [183]. Native ginsenosides show very limited absorption, and plasma concentrations are frequently insufficient to elicit consistent pharmacological activity [184]. Although the influence of the gut microbiota on drug metabolism, absorption, and disease progression is widely recognized [185], gastrointestinal biotransformation routes remain incompletely defined, and hepatic metabolic mechanisms are less systematically characterized [184].

Overall, ginsenosides are typically characterized by low water solubility, poor membrane permeability, and limited metabolic stability [186]. Deglycosylation and oxidation represent major metabolic routes with substantial hepatic involvement [186], while multiple studies suggest that key steps occur in the gastrointestinal tract, likely driven by microbial and digestive enzymatic degradation [187]. Elimination occurs mainly via bile, urine, and feces, with biliary and renal excretion playing important roles [186]. Chronic and subchronic toxicological studies using ginseng root extracts or individual ginsenosides generally support a favorable safety profile with low toxicity [188, 189], yet long‐term risk assessment across isoforms, formulations, and combination regimens remains warranted.

Collectively, these limitations hinder mechanistic elucidation and clinical translation. Future studies should prioritize systematic characterization of in vivo distribution, metabolism, and exposure, and advance delivery platforms—such as nanocarriers—to enhance bioavailability and therapeutic efficacy. Importantly, although existing evidence suggests that the inhibitory effects of ginsenosides in ovarian cancer may involve autophagy, it remains to be determined whether ginsenosides directly regulate autophagy and, if so, which specific autophagy nodes are targeted, thereby establishing a robust foundation for subsequent mechanistic validation and precision intervention.

Furthermore, many studies focus predominantly on nonselective autophagy, whereas selective autophagy has often been underappreciated [81]. Emerging evidence indicates that ER‐phagy and mitophagy can contribute to cancer development [82, 102], offering an additional layer to reconcile apparently conflicting observations. We therefore hypothesize that the anti‐ovarian‐cancer effects of ginsenosides may involve selective autophagy networks rather than solely altering bulk autophagy. Accordingly, future evaluations of autophagy should move beyond LC3‐II changes alone and incorporate receptor–substrate profiling (e.g., p62/SQSTM1, FAM134B) together with flux validation to improve interpretability and comparability. Mechanistic clarification should proceed in parallel with robust safety and efficacy verification to establish a stronger foundation for clinical application.

To address the existing gaps in the current understanding and therapeutic applications of ginsenosides in ovarian cancer, future studies should incorporate more specific research directions and detailed experimental methodologies. First, research should focus on identifying the isoform‐specific mechanisms of action of ginsenosides and their interactions with distinct autophagy‐related pathways. This includes developing robust models that simulate different ovarian cancer stages and tumor microenvironments, which will provide a clearer understanding of how ginsenosides affect autophagy and tumor progression in a context‐dependent manner. Second, advancing drug delivery strategies is crucial to improving ginsenosides' bioavailability and targeting efficiency. Nanoparticle‐based delivery systems, micellar formulations, and polymer‐drug conjugates should be explored in more detail to enhance tissue‐specific accumulation, reduce off‐target effects, and improve therapeutic efficacy. Furthermore, integrating ginsenosides with existing chemotherapy or immunotherapy regimens could open new avenues for combination therapies, potentially overcoming the limitations of conventional treatments. Finally, the role of selective autophagy in ginsenoside‐mediated therapy needs more focused attention. Future studies should employ advanced techniques, such as CRISPR‐Cas9‐based gene editing and fluorescence microscopy, to explore the mechanisms behind selective autophagy in response to ginsenosides and validate the findings through clinical trials. By addressing these specific research directions and translating them into clinical practice, the therapeutic potential of ginsenosides in ovarian cancer can be fully realized.

In summary, ginsenosides hold promise as multi‐target modulators of autophagy with potential to inhibit ovarian cancer progression, yet their net benefit is likely highly context‐dependent. Key priorities include (i) defining isoform‐specific direct targets and step‐specific effects on autophagic flux, (ii) systematically characterizing in vivo distribution, metabolism, and exposure while optimizing delivery systems to improve bioavailability and lesion accumulation, and (iii) integrating selective autophagy biomarkers with clinically relevant stratification strategies to enable a closed‐loop path from mechanism to translation.

## Author Contributions

Huiqin Liu proposed the concept and obtained funding support. Yuxin Guo expanded on the idea and wrote the manuscript. Cuilan Yun, Yuemei Zhang, and Xu Yang provided valuable suggestions for improving the content of the article and wrote a part of the manuscript.

## Funding

This work was supported by Science and Technology Program of the Joint Fund of Scientific Research for the Public Hospitals of Inner Mongolia Academy of Medical Sciences, 2024GLLH0463.

## Conflicts of Interest

The authors declare no conflicts of interest.

## Data Availability

Data sharing is not applicable to this article as no data sets were generated or analyzed during the current study.
